# Evaluation of a Possible Synergistic Effect of Meglumine Antimoniate with Paromomycin, Miltefosine or Allopurinol on in Vitro Susceptibility of *Leishmania tropica* Resistant Isolate

**Published:** 2013

**Authors:** Tahereh REZAEI RIABI, Iraj SHARIFI, Akram MIRAMIN MOHAMMADI, Ali KHAMESIPOUR, Maryam HAKIMI PARIZI

**Affiliations:** 1 Leishmaniasis Research Center, Kerman University of Medical Sciences, Kerman, Iran; 2 Center for Research &Training in Skin Diseases & Leprosy, Tehran University of Medical Sciences, Tehran, Iran

**Keywords:** In vitro, Treatment, *Leishmania tropica*, Resistance

## Abstract

**Background:**

Pentavalent antimonials are still the first choice treatment for leishmaniasis, but with low efficacy and resistance is emerging. In the present study, the effect of meglumine antimoniate (MA, Glucantime) combined with paromomycin, miltefosine or allopurinol on *in vitro* susceptibility of *Leishmania tropica* resistant isolate was evaluated.

**Method:**

The drugs were obtained from commercial sources and diluents of each drug in medium were prepared on the day of experiment. J774 A.1 murine macrophage cell lines were attached to the cultured on slide and incubated at 37 °C with 5% CO_2_ for 24 h. Then the stationary phase promastigotes were added to the cells and after 4 hrs of incubation different concentrations of MA, paromomycin, miltefosine or allopurinol were added and incubated for an additional of 72 h. Then the slides were dried and fixed with methanol, stained by Giemsa and studied under a light microscope. Drug activity was evaluated by assessing the macrophage infection rate and the number of amastigotes per infected macrophage was done by examining 100 macrophages. The experiment was done in triplicates.

**Result:**

Various concentrations of MA along with paromomycin, miltefosine or allopurinol significantly inhibited (*P*<0.01) the proliferation of *L. tropica* amastigote stage in the macrophage cell line as compared with MA alone or positive control.

**Conclusion:**

Combination of Glucantime with paromomycin, miltefosine or allopurinol showed a synergistic effect on the clinical isolate of *L. tropica in vitro*. Use of combination therapy is a new hope and a logical basis for therapy of the patients with cutaneous leishmaniasis. Further investigations are needed to evaluate the therapeutic effects of these drugs on the CL patients.

## Introduction

Leishmaniasis (CL) is caused by *Leishmania* spp and transmitted by various species of sandfly (1). CL is still a significant public health problem in endemic areas particularly in Iran (2). It is a major health problem in endemic areas of Iran (3). Leishmanaisis treatment and control are difficult because of the variety of epidemiological and clinical forms, different species of animal reservoirs and biological diference insand fly vectors (4). Available treatments are associated with adverse effects and are fairly effective (4). Although vaccination seems to be the most feasible and cost effective method for control of the disease currently there is no vaccine available (5).

At present, treatment of leishmaniasis in endemic areas is mainly restricted to chemotherapy (6). The first-line drug for CL in Iran is meglumine antimoniate (MA, Glucantime) but the efficacy is often questioned and resistance is emerging (7). Emphasis is presently focused on application of combination therapy strategies. Therefore, there is an urgent need for more effective with less duration therapeutic regimens and development of new treatment protocols. Combination therapy with MA and the second-line drugs including paromomycin, miltefosine or allopurinol is the only hope.

This study was aimed to evaluate various concentrations of meglumine antimoniate in combination with either paromomycin, miltefosine or allopurinol compared with each drug alone or positive control on in vitro susceptibility of *L. tropica* resistant isolate using an amastigote-macrophage model.

## Materials and Methods

### Parasite and macrophage culture

Macrophage cell line J774A.1 (ECACC number 91051511) was purchased from Pasture Institute,Tehran, Iran. Macrophages were kept in the laboratory by cryopreservation in liquid nitrogen and then by successive subcultures in RPMI- 1640 medium, supplemented with 15% heat-inactivated fetal calf serum (FCS), 1% penicillin and streptomycin (200µg/ml).Viability test using macrophage cell line and clinical stage of parasite (amastigotes) was performed by adding 90µl of trypan blue solution (0.2%) in saline solution containing 0.01% sodium aside to 10µl of cell suspension (10^6^ cells/ml). After 2 minutes, the cells were counted under a light microscope and viability was assessed as follows:

Viability= live cells / all counted cells × 100

Resistant isolate to meglumine antimoniate was recovered from a CL patient in Bam, southeastern Kerman province of Iran. This resistant isolate was detected by nested-PCR as *L. tropica* and further identified by conventional PCR for MDR1 gene (8). Subsequently the DNA extract was sequenced and recorded in Gene Bank under HM854717 Accession Number.

### Drugs

Meglumine antimoniate (MA), paromomycin, miltefosine and allopurinol were obtained from commercial sources. All drugs dilutions were prepared in RPMI- 1640 medium fresh on the day of assay. Various concentrations of MA, paromomycin, miltefosine or allopurinol alone or MA in combination with either paromomycin, miltofosine or allopurinol as compared with positive control were prepared. In the first combination, the concentration of MA remained constant while other drugs were used in decreasing order of concentrations. While, in the second combination, the concentrations of other drugs were constant and MA was used in a decreasing order of concentrations. For each amastigote assay 200µl of J774 A.1 murine macrophage cell lines (10^6^ cells/ml) were attached to the 8-chamber slide(Lab- Tek, Nalge Nunc International NY, USA), and incubated at 37 °C, with 5% CO_2_ for 24 hrs. Then the promastigotes in stationary phase were added to the macrophages and incubated for 24 hrs at different concentration of MA in combination with paromomycin, miltefosine or allopurinol and incubated for additional 72 h. Then the slides were dried, fixed with methanol, stained by Giemsa and studied under a light microscope. Drug activity was evaluated by two criteria; first, the mean infection rate of 100 macrophages and the second, the number of amastigotes in the macrophages by examining 100 macrophages. Every experiment was repeated three times.

## Results

The effect of MA, paromomycin, miltefosine or allopurinol alone on *in vitro* susceptibility of *L. tropica* resistant isolate (Table 1) showed that various concentrations of the drugs inhibited the growth of amastigotes in each macrophage as compared to the control group (*P*<0.01). However, the most effect was observed at concentration of 100µg/ml for each drug. In the second step, the effect of a constant concentration of MA coupled with either paromomycin, miltefosine or allopurinol was assessed on the same resistant isolate (Table 2). The finding indicated that different concentration of combined drugs inhibited more significantly than each drug alone the mean number of infected macrophages and the mean number of amastigotes per each macrophage in comparison to the control group (*P*<0.01).

**Table 1:** Comparative evaluation of the effect of meglumine antimoniate (MA), paromomycin (Paro), miltefosine (Milt) or allopurinol (Allo) alone on *in vitro* susceptibility of *Leishmania tropica* resistant isolate

Drugs	MA (µg/µl)		Paro (µg/µl)		Milt (µg/µl)		Allo (µg/µl)	
Concentration	No. of infected macrophage ±SD	No. of amastigote ±SD	No. of infected macrophage ±SD	No. of amastigote ±SD	No. of infected macrophage ±SD	No. of amastigote ±SD	No. of infected macrophage ±SD	No. of amastigote ±SD
**0.00(Control)**	65.33±2.58	104.67±5.58	65.33±2.517	104.67±5.508	65.33±2.58	104.67±5.51	65.33±2.52	104.67±5.51
**6.25**	64.00±2.00	96.00±5.29	62.33±2.082	101.33±3.215	77.67±6.66	114.33±8.1	64.00±2.00	96.00±5.29
**12.50**	57.67±3.51	76.00±4.58	57.33±2.082	94.67±10.066	65.33±2.52	85.00±5.51	57.67±3.51	76.00±4.58
**25.00**	51.33±4.04	77.67±0.10	54.33±2.082	61.67±2.082	48.67±0.58	94.67±5.52	51.33±4.04	77.67±0.10
**50.00**	46.00±1.00	64.67±4.16	44.67±3.055	58.00±3.606	40.33±2.52	54.67±4.16	46.00±1.00	64.67±4.16
**100.00**	8.67±2.08	10.33±2.31	20.33±2.517	23.67±3.055	10.33±1.53	12.67±1.53	8.67±2.08	10.33±2.31

A significant difference between various concentrations of MA, Paro, Milt or Allo and positive control was observed (*P*< 0.01).

**Table 2:** Comparative evaluation of the effect of meglumine antimoniate(MA, 100 or 50 µg/ml) combined with various concentrations of paromomycin (Paro), miltefosine (Milt) or allopurinol (Allo) on *in vitro* susceptibility of *Leishmania tropica* resistant isolate

Drugs	MA+ Paro+Medium (µg/µl)		MA+ Milt+Medium (µg/µl)		MA+ Allo+Medium (µg/µl)	
Concentration	No of infected macrophage ±SD	No of amastigote ±SD	No of infected macrophage ±SD	No of amastigote ±SD	No of infected macrophage ±SD	No of amastigote ±SD
**0.00(Control)**	65.33±2.52	104.67±5.51	65.33±2.52	104.67±5.51	65.33±2.52	104.67±5.51
**100+100**	2.33±0.58	2.33±0.58	1.00±1.00	1.00±1.00	9.33±1.53	9.67±1.53
**50+50+100**	7.00±1.00	7.76±1.16	6.00±1.00	7.00±1.00	16.00±4.36	23.33±7.64
**50+25+125**	39.00±1.00	54.00±3.61	40.33±1.53	54.33±2.89	26.67±4.16	37.00±6.56
**50+12.5+137.5**	41.68±3.51	58.00±3.65	41.33±2.52	57.00±3.61	36.67±4.16	53.00±7.00
**50+6.25+143.75**	43.00±3.61	64.33±4.04	46.00±1.00	61.33±2.31	44.33±3.51	73.00±6.25

A significant difference between MA combined with various concentrations of Paro, Milt or Allo and positive control was observed (*P*< 0.01).

In the last series, variable concentrations of MA were coupled with each drug alone (Table 3). Again a significant effect (*P*<0.01) was observed similar to the constant concentrations of MA, when the infected macrophages and the mean number of amastigotes were evaluated.

**Table 3:** Comparative evaluation of the effect of paromomycin (100 or 50 µg/ml), miltefosine (100 or 50 µg/ml) or allopurinol (100 or 50 µg/ml) combined with various concentrations of meglumine antimoniate (MA) on *in vitro* susceptibility of *Leishmania tropica* resistant isolate

Drugs	Paro+ MA +Medium (µg/µl)		Milt+ MA +Medium (µg/µl)		Allo+ MA +Medium (µg/µl)	
Concentration	No. of infected macrophage ±SD	No. of amastigote ±SD	No. of infected macrophage ±SD	No. of amastigote ±SD	No. of infected macrophage ±SD	No. of amastigote ±SD
**0.00(Control)**	65.33±2.52	104.67±5.51	65.33±2.517	104.67±5.508	65.33±2.52	104.67±5.51
**100+100**	1.00±1.00	1.00±1.00	0.67±1.555	0.67±1.555	6.00±1.00	6.33±1.53
**50+50+100**	7.67±1.53	11.00±1.00	9.00±2.000	10.33±2.517	14.00±4.00	21.33±5.86
**50+25+125**	33.00±2.00	46.67±3.79	42.33±1.528	63.00±2.646	28.00±4.16	49.33±6.56
**50+12.5+137.5**	37.33±1.53	55.00±3.61	50.00±2.646	83.00±4.000	36.00±2.65	58.33±1.16
**50+6.25+143.75**	46.33±2.52	83.00±5.13	57.33±3.055	97.00±5.292	45.67±3.51	60.67±3.06

A significant difference between Paro, Milt or Allo combined with various concentrations of MA and positive control was observed (*P*< 0.01)

## Discussion

Leishmaniasis continues to be an important public health challenge in endemic countries (4). The treatment of choice for this disease has long been pentavalet antimony compounds such as meglumine antimoniate (Glucantime) and sodium stibogluconate (Pentostam). These drugs are no longer effective in most tropical and sub-tropical countries where the two anthroponotic leishmaniasis (ACL and AVL) due to *L. tropica* and *L. donovani* are present (8, 9). Resistance to these drugs have emerged in many foci and widely spread throughout the endemic areas (6). The main problem to the success of combination treatment in preventing the emergence of resistance will be inadequate treatment including incorrect dosing, sub-standard drugs, poor adherence and unusual pharmacokinetics (10).

Combination treatments were highly effective and really could make a major contribution to global leishmaniasis control at *in vitro* level (11). The theory underlying combination drug treatment for most infectious diseases such as HIV/AIDES, malaria, leprosy and tuberculosis is now well known, and the same general principle is now widely accepted for leishmaniasis. If two drugs are used with different modes of actions, then they could helpfully prevent effectively emergence of the new resistant mutant (12).

Hopefully the application of pentavalent antimonials including meglumine antimoniate as the first-line of treatment along with a second-line drug such as paromomycin, miltefosine or allopurinol with different modes of action would synergistically reduce the number of *Leishmania* parasite, inhibiting the proliferation of the clinical stage within each macrophage and eventually limits the burden of the disease (6). Although the effect of MA in combination with each drug was significantly reduced, the two indices related to the amastigote-macrophage model, however, the combination of MA along with paromomycin showed the highest effect, followed by MA plus miltefosine or MA coupled with allopurinol.

## Conclusion

Since combination effect on the clinical stage in an amastigote- macrophage model, it could be a new hope and a logical basis for therapy of the patients with CL. Further studies are required to evaluate the therapeutic effects of these drugs on the CL patients in endemic countries.
